# The MicroRNA Landscape of MYCN-Amplified Neuroblastoma

**DOI:** 10.3389/fonc.2021.647737

**Published:** 2021-05-07

**Authors:** Danny Misiak, Sven Hagemann, Jessica L. Bell, Bianca Busch, Marcell Lederer, Nadine Bley, Johannes H. Schulte, Stefan Hüttelmaier

**Affiliations:** ^1^ Institute of Molecular Medicine, Martin Luther University Halle-Wittenberg, Halle, Germany; ^2^ Department of Pediatric Oncology and Hematology, Charité-Universitätsmedizin Berlin, Berlin, Germany; ^3^ German Consortium for Translational Cancer Research (DKTK), Partner Site Charité Berlin, Berlin, Germany

**Keywords:** microRNAs, MYCN, IGF2BP1, mir-17-92 cluster, diagnostic marker, neuroblastoma, pediatric cancer

## Abstract

*MYCN* gene amplification and upregulated expression are major hallmarks in the progression of high-risk neuroblastoma. MYCN expression and function in modulating gene synthesis in neuroblastoma is controlled at virtually every level, including poorly understood regulation at the post-transcriptional level. MYCN modulates the expression of various microRNAs including the miR-17-92 cluster. MYCN mRNA expression itself is subjected to the control by miRNAs, most prominently the miR-17-92 cluster that balances MYCN expression by feed-back regulation. This homeostasis seems disturbed in neuroblastoma where *MYCN* upregulation coincides with severely increased expression of the miR-17-92 cluster. In the presented study, we applied high-throughput next generation sequencing to unravel the miRNome in a cohort of 97 neuroblastomas, representing all clinical stages. Aiming to reveal the *MYCN*-dependent miRNome, we evaluate miRNA expression in *MYCN*-amplified as well as none amplified tumor samples. In correlation with survival data analysis of differentially expressed miRNAs, we present various putative oncogenic as well as tumor suppressive miRNAs in neuroblastoma. Using microRNA trapping by RNA affinity purification, we provide a comprehensive view of MYCN-regulatory miRNAs in neuroblastoma-derived cells, confirming a pivotal role of the miR-17-92 cluster and moderate association by the let-7 miRNA family. Attempting to decipher how *MYCN* expression escapes elevated expression of inhibitory miRNAs, we present evidence that RNA-binding proteins like the IGF2 mRNA binding protein 1 reduce miRNA-directed downregulation of MYCN in neuroblastoma. Our findings emphasize the potency of post-transcriptional regulation of *MYCN* in neuroblastoma and unravel new avenues to pursue inhibition of this potent oncogene.

## Introduction

Neuroblastoma, the most common extracranial solid childhood tumor, originates from precursors of the sympathetic nervous system and account for approximately 15% of all cancer-related death in infants (1). The clinical presentation of neuroblastoma is remarkably heterogeneous in pathological, genetic and biological characteristics, ranging from spontaneous regression or differentiation of the tumor to a high-risk aggressive disease. Neuroblastoma is thought to arise from sympathoadrenal lineage precursor cells, derived from the neural crest. Most frequently tumor initiation emanates from one of the adrenal glands, but is also observed in the neck, chest, abdomen or along the spine (2). Risk classification depends on several clinical and biological factors, such as age at diagnosis, stage, histology and genetic aberrations (2). Favorable neuroblastoma, in particular clinical stage 4S neuroblastoma often undergo remission without any therapy. In contrast, the clinical outcome of patients with high-risk neuroblastoma stagnates despite many therapeutic approaches like surgery, radiation, chemotherapy, stem cell transplantation or immunotherapy (2). The 5-year survival rate of high-risk patients is still under 50% and the treatment remains challenging (3).

The *MYCN* oncogene is frequently amplified in high-risk neuroblastoma and a known biomarker for disease stratification (4). Amplification or severe upregulation results in a lower survival probability and a more aggressive disease. Regulation of MYCN mRNA is not fully understood, but post-transcriptional regulation by microRNAs (miRNAs) seems to be important (5, 6). Some MYCN-targeting miRNAs are downregulated in neuroblastoma like for instance members of the let-7 family. This, at least partially, is due to the increased expression of LIN28B, which impairs let-7 biogenesis and promotes neuroblastoma formation (7–9). Hence, it was assumed that upregulated expression of MYCN protein is supported by the overall downregulation of MYCN-inhibitory miRNAs in neuroblastoma (10). However, various miRNAs impairing MYCN expression, in particular members of the miR-17-92 cluster are substantially transcriptionally activated by MYC/MYCN protein and consequently co-upregulated with *MYC/MYCN* in various malignancies. This suggests that this negative feed-back regulation is at least partially uncoupled in *MYCN*-amplified (MNA) neuroblastoma, either due to a miRNA decoy function of the MYCN-3’UTR, proposed for the let-7a miRNA (11), or by trans-acting factors like RNA-binding proteins (RBPs). In respect of the latter, ELAVL4 (Embryonic lethal, Abnormal Vision, Drosophila-Like 4) RNA-binding protein (also termed HuD) and the IGF2 mRNA binding protein 1 (IGF2BP1) are of immediate interest. ELAVL4 was proposed to antagonize downregulation of MYCN expression by miR-17 (12). Expression of the oncofetal IGF2BP1 is upregulated in MNA neuroblastoma (13, 14), promotes MYCN protein and RNA expression (13), and is considered a potent and conserved inhibitor of miRNA-dependent downregulation of oncogenic factors in cancer (15, 16).

In the presented study, we provide a comprehensive analysis of miRNA expression in 97 neuroblastoma samples and reveal signatures of significantly upregulated as well as decreased miRNAs in MNA neuroblastoma. On the basis of miRNA trapping by RNA affinity purification [miTRAP, (17)], we confirm and report novel MYCN-regulatory miRNAs expressed in MNA neuroblastoma as well as *MYCN*-driven glioblastoma cell models. Finally, we evaluate if trans-acting factors, in particular IGF2BP1 and ELAVL4, or a putative miRNA decoy function of the MYCN-3’UTR uncouple increased expression of MYCN-regulatory miRNAs in neuroblastoma from elevated expression of MYCN protein. Our findings provide strong evidence that enforced expression of MYCN in MNA neuroblastoma essentially relies on IGF2BP1.

## Materials and Methods

### Small RNA Library Construction, High-Throughput Sequencing and Differential Expression Analysis

Total RNA of neuroblastoma tumor samples [provided by Cologne/Essen neuroblastoma tumor bank upon application, as indicated in Bell et al. (13); for tumor information see **Supplementary Table 6**] was extracted from 30 µg of primary tumor tissue using the Qiagen ALLprep tumor protocol with miRNeasy kits (Qiagen). 500 ng of total RNA was used in the small RNA protocol with the TruSeq™ Small RNA sample prepkit v2 (Illumina) according to the instructions of the manufacturer. The barcoded libraries were size restricted between 140 and 165 bp, purified and quantified using the Library Quantification Kit–Illumina/Universal (KAPA Biosystems) according to the instructions of the manufacturer. A pool of 10 libraries was used for cluster generation at a concentration of 10 nM using an Illumina cBot. Sequencing of 51 bp was performed with an IlluminaHighScan-SQ sequencer at the sequencing core facility of the IZKF Leipzig (Faculty of Medicine, University Leipzig) using version 3 chemistry and flowcell according to the instructions of the manufacturer. Demultiplexing of raw reads, adapter trimming and quality filtering were done according to Stokowy et al. (18). Unstranded single-end reads with 51 bp in length were trimmed for adapter and low quality sequences using Cutadapt (v 1.18). Trimmed reads were mapped to the human genome (hg38 UCSC) using Bowtie2 [v 2.2.6, (19)], allowing for one mismatch in the seed region (parameter-N 1). Subsequent, mapped reads were summarized using featureCounts [v 1.6.3, (20)] and miRBase [v 22, (21)] annotations. Differential expression of miRNAs was determined using R package edgeR [v 3.28, (22)] utilizing trimmed mean of M-values [TMM, (23)] normalization of read counts. A false discovery rate (FDR) value below 0.05 was considered as threshold for the determination of differential expression.

### Survival Analysis

Survival analyses were performed using differentially expressed miRNAs (*MYCN*-amplified (MNA) *vs.* none *MYCN*-amplified (nMNA)) by use of TMM normalized expression data processed as mentioned in *Small RNA Library Construction, High-Throughput Sequencing and Differential Expression Analysis*. These miRNAs were associated with available clinical data obtained from the analyzed tumor cohort. The log-rank test was implemented in an R-script according to the description in Bewick et al. (24). High and low expression groups were separated by the respective median of normalized miRNA expression values. Hazardous ratios (HR) for differential expressed miRNAs were determined by Kaplan–Meier plotting using median cut-off. For determining the overall HR of oncogenic and tumor suppressive miRNA signatures respectively, the log_2_-transformed expression values of all oncogenic or tumor suppressive miRNAs were first median-centered, subsequently combined and then divided into high and low expression group (median cut-off).

### Identification of Putative Oncogenic and Tumor Suppressive miRNAs

We identified miRNAs for a classification of putative oncogenic (upregulated in MNA, poor prognostic value) and tumor suppressive (downregulated in MNA, good prognosis) miRNAs in a MYCN dependent manner. This identification is based on the aforementioned results from differential gene expression (see *Small RNA Library Construction, High-Throughput Sequencing and Differential Expression Analysis*), comparing MNA against nMNA tumors, in combination with the prognostic values from the survival data analysis (see *Survival Analysis*). In more detail, miRNAs were first abundance filtered (average CPM ≥1, counts per million mapped reads) and checked for significant differential expression changes between MNA *vs.* nMNA (FDR <0.05). Subsequent, miRNAs were selected by significant difference in survival by hazard ratios (HR below or above 1) with a log-rank p-value below 0.05. Finally, miRNA duplicates (miRNAs mapped to different chromosomal locations) were removed by selecting miRNAs with most significant FDR from differential expression analysis.

### MicroRNA–Target Predictions

Predicted and validated miRNA-MYCN bindings were obtained by utilizing the R-package multiMiR [v1.12.0, database version 2.3.0, (25)]. Eight databases containing predicted and two databases including validated binding information were queried (prediction cutoff 20%) for targeting MYCN mRNA. If a certain miRNA–mRNA pair was obtained by at least two (predicted) and one (validated) database, it was considered as a putative interacting pair (**Supplementary Table 3**).

### MiTRAP Experiments

MiTRAP experiments using 3´UTR of MYCN or MS2 control RNA were essentially performed as described recently (17). Purified RNA was sent for short-read RNA sequencing. Single-end sequencing was performed on Illumina HiSeq 1500 platform at Novogene (Hong Kong). Originating sequence reads of 50 bp in length were quality checked [FastQC, v. 0.11.8]. Adapters and low quality read ends were clipped off, resulting in reads with a length of 16–50 bases. Kept reads were further processed as described in *Small RNA Library Construction, High-Throughput Sequencing and Differential Expression Analysis* to determine normalized read counts. Fold enrichment was achieved by comparing miRNAs in the MYCN-3’UTR pulldown to MS2 control pulldown.

### Western Blotting

Western blots were analyzed by an Odyssey Infrared Imaging System (LI-COR Biosciences). Antibodies used included anti-AGO2 (clone 11A9, serum was provided by Prof. Dr. Gunther Meister, dilution 1:5 in 5% BSA), anti-VCL (Sigma, V9131, dilution 1:5,000 in 5% BSA), anti-MBP (Cell Signaling, E8032, dilution 1:1,000 in 5% BSA) and IRDye 680/800CW-labeled mouse or rat secondary antibodies (LI-COR Biosciences, dilution 1:10,000 in 5% BSA).

### Plasmids and Cloning

Cloning strategies including vectors, oligonucleotides used for PCR and restrictions sites are summarized in **Supplementary Table 5**. All constructs were validated by sequencing.

### Cell Culture and Transfection

BE(2)-C cells were cultured in a 1:1 mixture of DMEM/F12 (with HEPES, Gibco) and EMEM (ATCC) supplemented with 10% FBS. KNS42 cells were cultured in DMEM (Gibco) supplemented with 10% FBS. Cells were grown at 37°C and 5% CO_2_. 3.5 × 10^5^ BE(2)-C cells were seeded in a 6-well plate and after 6 h transiently transfected with 2.5 µg GFP reporter and 7 µg iRFP vector using 14.5 µl Lipofectamine 3000 reagent (Life Technologies, 19 µl P3000 reagent, 125 µl OPTImem (Gibco)). Medium was changed after 24 h and cells were harvested and analyzed by flow cytometry 48 h post transfection.

### Flow Cytometry Analysis

GFP and iRFP fluorescence was measured with a MACSQuant system. Transfected cells were harvested, washed once with PBS and then resuspended in 1% BSA in PBST. To exclude dead cells DAPI was added to the sample by the machine. Cells were gated to analyze a homogenous and single cell population. Mean fluorescence of GFP and iRFP double positive cells was analyzed. For **Figures 4B** and **5C** each GFP reporter (**Figure 3A**) was co-transfected with respective iRFP vectors (**Figures 4A**, **5A**). The mean GFP fluorescence of empty GFP, GFP-MYCN 3´UTR WT and GFP-MYCN 3´UTR mut transfection was first normalized to respective empty iRFP vector co-transfection and then normalized to empty GFP with respective iRFP vector transfection. For **Figures 5B** the mean iRFP fluorescence was normalized to empty iRFP vector co-transfection.

## Results

### Deregulated miRNA Expression Distinguishes MNA Neuroblastoma

**Figure 1:**
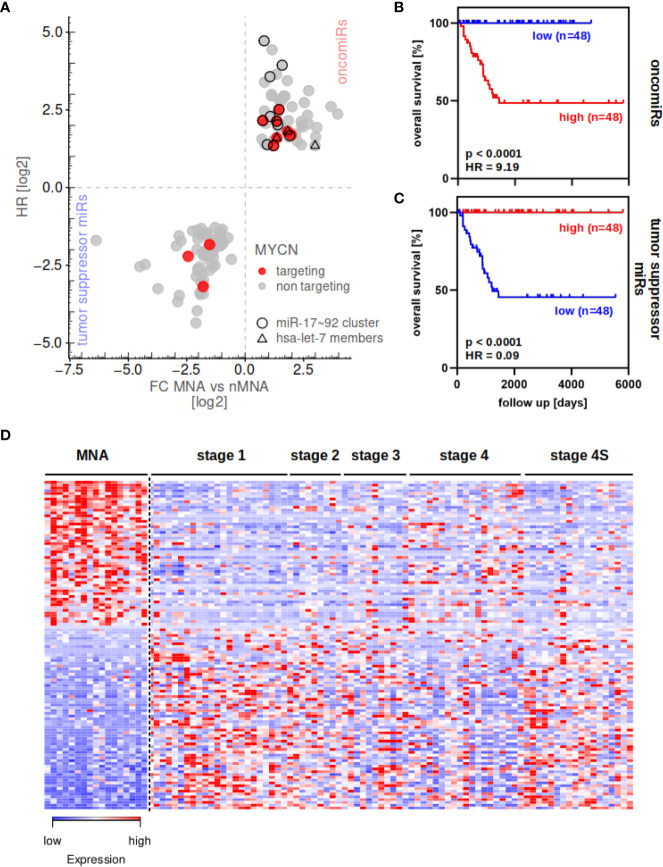
miRNA expression can distinguish between MYCN-amplified and non-amplified tumors. **(A)** Differential miRNA expression analysis in 17 MNA and 80 nMNA neuroblastoma tumors and subsequent determination of hazardous ratio (HR, one sample lack survival data) of these miRNAs revealed 52 oncogenic and 66 tumor suppressive miRNAs (see **Supplementary Table 1** for fold changes and HR). MYCN-targeting miRNAs are indicated by colors, members of the miR-17-92 cluster and let-7 family are indicated by circles and triangles, respectively. Survival analysis of upregulated **(B)** or downregulated **(C)** miRNA signature show strong association of the respective miRNAs to overall patient survival probability. **(D)** The 118 differential expressed miRNAs distinguish MNA and nMNA tumors, as indicated by heatmap. Expression values are scaled for individual miRNAs (rows in heatmap). MYCN-amplified tumors (MNA) as well as clinical staging of nMNA tumors are indicated in the top panel.

The miRNA transcriptome was profiled in 97 primary neuroblastoma tumors, including 17 *MYCN*-amplified (MNA) and 80 none *MYCN*-amplified (nMNA) tumors and differential expression of miRNAs between these two groups was investigated (for tumor information see **Supplementary Table 6**). This analysis indicated 52 significantly up- and 66 significantly downregulated miRNAs in MNA when compared to nMNA tumors (FDR <0.05, **Figure 1A**). Among these, we observed seven upregulated miRNAs reported to control MYCN mRNA expression, but only three MYCN-targeting miRNAs downregulated in MNA (**Figure 1A**, red). To generally investigate to what extent a low or high expression of these miRNAs affects the overall survival in neuroblastoma, we applied Kaplan–Meier survival analysis to these miRNA signatures upregulated and downregulated in MNA neuroblastoma. By dividing the cohort into low and high expressing groups (see *Materials and Methods*), these analysis confirmed that high expression of upregulated miRNAs was associated with substantially reduced overall survival probability, as expected and indicated by a Hazard ratio (HR) of 9.19 (**Figure 1B**). The opposite was observed for miRNAs significantly decreased in MNA tumors, for which low expression was associated with an overall poor prognosis (**Figure 1C**; HR = 0.09). Notably, the investigation of the overall prognosis relevance of each differentially expressed miRNA confirmed a rather oncogenic role, indicated by HR values greater 1, for miRNAs upregulated in MNA. Rather tumor suppressive functions (HR <1) were indicated for miRNAs downregulated in MNA (**Figure 1A**, **Supplementary Table 1**). Accordingly, miRNAs upregulated in MNA were considered to indicate oncogenic (oncomiRs), whereas miRNAs decreased in MNA indicated tumor-suppressive miRNAs, respectively. In agreement with these analyses, the investigation of miRNA expression in individual tumor samples clearly indicated that MNA tumors are distinguished from nMNA neuroblastoma by a severe upregulation of oncomiRs (red in MNA) and downregulation of tumor suppressive miRNAs (blue in MNA), as depicted by a heat map (**Figure 1D** and **Supplementary Table 2**).

In contrast to previous reports, suggesting downregulation of MYCN-regulatory miRNAs in *MYCN*-driven neuroblastoma (10), we observed a variety of known MYCN-targeting miRNAs among oncomiRs associated with *MYCN* amplification. Most prominently, members of the miR-17-92 cluster, of which miR-17/-18a/-19a/-20a/-25/-92a/-92b and miR-93 were significantly upregulated in MNA neuroblastoma, were among oncomiRs. This is concise with the role of MYC and MYCN protein in promoting expression of this miRNA cluster *via* E-box sequences in the promotor region upstream of the miR-17-92 cluster (26, 27). In support of various previous studies, we furthermore observed upregulation of miR-9 (28), miR-15b-5p (29), miR-16-2-3p (30), and miR-181a/b (27) in MNA tumors. Likewise, we confirmed previously reported downregulation of miRNAs in MNA tumors, including miR-628-5p, miR-137-3p, miR-542-5p and miR-488-5p (29–31). In addition, we identified various previously none reported upregulated and downregulated miRNAs in MNA (see **Supplementary Table 1**). Surprisingly, miR-34c expression was elevated in MNA tumors, although previous studies claimed that the MYCN-targeting miR-34 family is downregulated in high-risk neuroblastoma (32–34). In sum our findings suggested that a variety of miRNAs reported to control MYCN mRNA expression are upregulated in MNA. To corroborate these findings, we re-analyzed the expression of miRNAs predicted to target MYCN mRNA in neuroblastoma. To this end, candidate regulatory miRNAs controlling MYCN expression were identified by investigating eight miRNA prediction databases (**Supplementary Table 3**). In support of the notion that MYCN-regulatory miRNAs are rather downregulated in *MYCN*-driven neuroblastoma (10), the reported miR-542 (35), and the predicted MYCN-regulatory miR-488 were identified among miRNAs downregulated in MNA tumors. In contrast, however, the vast majority of reported MYCN-controlling miRNAs were found to be upregulated in MNA tumors. These included miR-17 (12, 36), miR-19a (5), as well as miR-15b and miR-16 (37). Furthermore miR-20a and miR-93 (same seed sequence as miR-17) and members of the let-7 family, expected or reported to control MYCN mRNA expression, were among miRNAs upregulated in MNA tumors.

### MiTRAP Reveals MYCN-Regulatory miRNA Candidates

**Figure 2:**
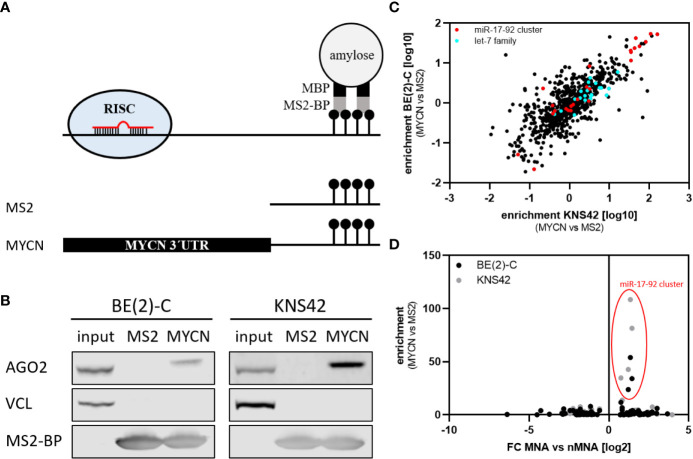
miTRAP identified selective co-purified miRNAs with *in vitro* transcribed bait RNA. **(A)** Scheme of the miTRAP procedure. *In vitro* transcribed bait RNAs comprising two MS2 stem-loops fused to the 3´ end of bait transcripts were immobilized on amylose resin (light gray) *via* recombinant MBP-fused (black) MS2-BP (dark gray) protein (upper panel). Scheme of the used bait RNAs (lower panel). MS2: 120-nt-long control RNA encoded by the template vector, MYCN: wild type 3´UTR of the MYCN mRNA. **(B)** Western blot analysis of indicated proteins isolated from BE(2)-C and KNS42 cells co-purified with MS2 control transcript (MS2) or the MS2-fused MYCN 3´UTR (MYCN), respectively. Vinculin (VCL) served as negative control for unspecific binding, whereas MS2-BP indicates equal loading of the resin. **(C)** Enrichment of specific miRNAs was calculated as ratio of MYCN 3´UTR compared to MS2 control pulldown. Enrichment from BE(2)-C and KNS42 cells show strong consistency in enriched miRNAs (Pearson correlation: R_P_ = 0.7784, p < 0.0001). The miR-17-92 cluster is depicted in red, the let-7 family in cyan. **(D)** The enrichment of miRNAs with the MYCN-3’UTR in BE(2)-C and KNS42 cell lines were compared to the fold change of miRNA expression in MNA versus nMNA tumors. Only members of the miR-17-92 cluster are consistently and substantially enriched at the MYCN 3´UTR and upregulated in MNA tumors.

Our investigation of miRNA expression in MNA neuroblastoma indicated that a substantial number of reported or predicted MYCN-regulatory miRNAs are upregulated in MNA. To evaluate MYCN-targeting directly, we employed miTRAP (miRNA trapping by RNA *in vitro* affinity purification) using the MYCN-3’UTR as bait to identify associating miRNAs in the *MYCN*-amplified BE(2)-C neuroblastoma and *MYCN*-driven KNS42 glioblastoma cell lines [**Figure 2A**, (17)]. KNS42 cells were included to address conserved association of miRNAs. The affinity purification of the MS2-fused MYCN-3´UTR by MS2-binding protein (MS2-BP) resulted in a robust co-purification of the RISC protein AGO2 in both cell lines, whereas neither vinculin (VCL, negative control) nor AGO2 were co-purified with the MS2 bait control (**Figure 2B**). The association of miRNAs was investigated by determining miRNA abundance using small RNA sequencing of MYCN-3’UTR and MS2 control pulldown fractions in three independent analyses. Enrichment of miRNAs was determined by the fold enrichment of miRNAs in the MYCN-3’UTR pulldown compared to MS2 controls. The comparison of miRNA enrichment revealed a striking conservation of miRNA co-purification in both cell lines (**Figure 2C** and **Supplementary Table 4**; R_P_ = 0.7784, p <0.0001). In contrast, miRNA enrichment was largely independent of miRNA abundance (**Supplementary Figure S1**), as indicated by Pearson correlation coefficient of R_P_ = −0.001072 9BE(2)-C, p = 0.96880 and R_P_ = −0.002382 (KNS42, p = 0.9306). Among the most severely enriched miRNAs were members of the MYCN-regulatory miR-17-92 cluster (**Figure 2C**, red), e.g. miR-17, as well as other validated MYCN-regulatory miRNAs like miR-29 (38). Notably, among the top 15 enriched miRNAs more than half belong to the miR-17-92 cluster (**Figure 2C**, red and **Supplementary Table 4**). Surprisingly, however, despite substantial abundance (1.15% (BE(2)-C) or 3.12% (KNS42) of all reads in input) and reported regulation of MYCN mRNA (39), let-7 family members were only modestly enriched with the MYCN-3’UTR (**Figure 2C**, cyan). Likewise, although reported to regulate MYCN expression (34), miR-34a was not enriched with the MYCN-3’UTR, presumably due to low abundance. Novel miRNAs potentially controlling MYCN mRNA expression due to substantial and conserved enrichment with the MYCN-3’UTR discovered by miTRAP are miR-193b-3p and the miR-302 family, both predicted as MYCN-regulatory miRNAs, as well as miR-6782-5p and miR-1248 (**Supplementary Table 4**).

The comparison of miRNA enrichment with the MYCN-3’UTR and altered expression of miRNAs in MNA neuroblastoma indicated that the miR-17-92 cluster miRNAs stood out due to substantially increased expression in MNA neuroblastoma and the obviously substantial enrichment by miTRAP in both analyzed cell lines (**Figure 2D**). In sum, the presented analyses provided further evidence that MYCN-regulatory miRNAs, most prominently the miR-17-92 cluster, are enriched in MNA neuroblastoma suggesting mechanisms that limit MYCN downregulation by miRNAs in this diseases subtype.

### IGF2BP1 Is a Potent, 3’UTR- and miRNA-Dependent Regulator of MYCN Expression

Our studies imply that elevated *MYCN* expression in MNA neuroblastoma involve mechanisms uncoupling *MYCN*-driven expression of miR-17-92 miRNAs from the inhibition of MYCN mRNA by feed-back regulation. To this end, we addressed the potential involvement of two RNA-binding proteins (RBPs), ELAVL4 and IGF2BP1. ELAVL4 (HuD) was reported to interfere with miR-17-directed inhibition of MYCN expression by associating with the MYCN-3’UTR (12, 36, 40). However, ELAVL4 mRNA expression is significantly downregulated in MNA neuroblastoma (**Supplementary Figure S2**). IGF2BP1 is an oncofetal RBP upregulated in MNA neuroblastoma and reported to control MYCN expression [(13, 14), **Supplementary Figure S2**]. Moreover, IGF2BP1’s main and conserved role in cancer cells is the impairment of miRNA-directed mRNA degradation by recruiting target mRNAs to miRNA/RISC-devoid mRNPs (15, 16).

**Figure 3:**
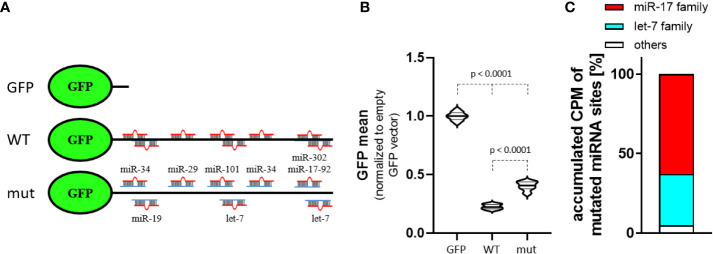
MYCN 3´UTR is a strong miRNA target. **(A)** Scheme of used GFP reporters. Control reporter (GFP) contains only a minimal, vector-encoded 3´UTR. MYCN wildtype (WT) and mutated (mut) 3´UTR were inserted 3’ of the GFP open reading frame. The mutated 3´UTR was generated by conversion of seed sequences in eight indicated miRNA-targeting sites. **(B)** Normalized mean GFP fluorescence in BE(2)-C cells transiently transfected with GFP reporters. The WT MYCN-3´UTR is strongly repressed, indicated by lower GFP fluorescence. Mutation of several miRNA-binding sites increases GFP fluorescence (n = 3). **(C)** Among the accumulated CPM (count per million) of mutated MYCN-regulatory miRNA sites in BE(2)-C the miR-17 seed family (red) and let-7 family (cyan) are the most abundant. Statistical significance was determined by Student’s t-test (p-values indicated).

To investigate MYCN-3’UTR dependent regulation in MNA neuroblastoma BE(2)-C cells, we employed a dual fluorescent reporter assay allowing the rapid assessment of altered protein expression by flow cytometry. In addition to the MYCN wild type 3’UTR fused to GFP (**Figure 3A**), we included a control reporter with a vector encoded 3’UTR (GFP) and a reporter in which targeting seeds of the miR-17-92, let-7 as well as some other MYCN-regulatory, indicated miRNAs were inactivated by mutation. In comparison to the control reporter, the activity of both reporters, either comprising the MYCN wild type 3’UTR or the mutated MYCN-3’UTR showed markedly reduced GFP expression (**Figure 3B**). This clearly indicates a pivotal role of the MYCN-3’UTR in controlling MYCN expression. Notably, the mutation of eight miRNA binding sites led to a significant increase of GFP expression. Thus miRNA-dependent regulation, in particular by the miR-17-92 cluster which summed up to ~63% of MYCN-regulatory miRNAs with inactivated seeds in the mutant reporter (**Figure 3C**), substantially contribute to the 3’UTR-dependent regulation of MYCN expression.

**Figure 4:**
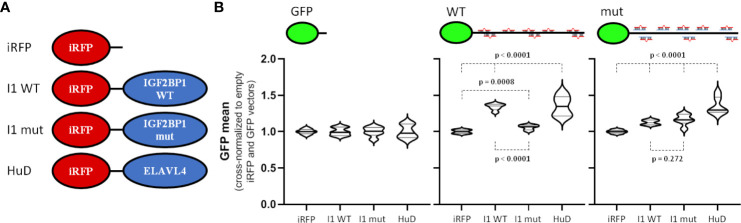
IGF2BP1 and ELAVL4 are potent, 3´UTR-dependent regulators of MYCN expression. **(A)** Scheme of used iRFP vectors. Control vector (iRFP) or vectors with iRFP-fused wildtype IGF2BP1 (I1 WT), RNA-binding deficient mutant IGF2BP1 (I1 mut) or ELAVL4 (HuD) were transfected with GFP reporters (see **Figure 3A**) into BE(2)-C cells. **(B)** Transfected BE(2)-C were analyzed by flow cytometry. GFP and iRFP double positive cells were analyzed to determine the influence of IGF2BP1 (WT and mut) as well as ELAVL4 to the MYCN 3´UTR upon cross-normalization (n = 3). Statistical significance was determined by Student’s t-test (p-values indicated).

To investigate the potential role of ELAVL4 and IGF2BP1 in the 3’UTR- and miRNA-dependent regulation of MYCN expression, the proteins were co-expressed with GFP reporters as iRFP-fused (near-infrared fluorescent protein) proteins (**Figure 4A**). In addition to wild type IGF2BP1, we also analyzed an RNA-binding deficient mutant protein (I1 mut). This failed to restore RNA-dependent regulation by IGF2BP1 (16, 41–43), but maintains RNA-binding independent regulation of the recently reported protein-directed activation of the SRC kinase (44). Activity of the GFP control reporter remained largely unaffected by the expression of iRFP-fused proteins when comparing mean GFP fluorescent (**Figure 4B**, left panel). On the contrary, expression of the GFP-reporter comprising the wild type MYCN-3’UTR was significantly elevated by co-expression of IGF2BP1 as well as ELAVL4 (**Figure 4B**, middle panel). Importantly, an only modestly increased GFP abundance was observed in cell co-expressing the RNA-binding deficient IGF2BP1. This suggested, that both, IGF2BP1 and ELAVL4, promote MYCN expression, as previously proposed, and that this regulation largely relies on the MYCN-3’UTR. Finally, we analyzed how inactivation of miRNA sites influences regulation by IGF2BP1 and ELAVL4 (**Figure 4B**, right panel). Although only modest, both tested IGF2BP1 proteins led to a slightly upregulated expression of the respective GFP-reporter. However, essentially no difference was observed between the wild type IGF2BP1 and RNA-binding deficient protein, suggesting secondary, RNA-binding independent regulation. Surprising was the essentially unaltered activation of the wild type and miRNA-mutated MYCN-3’UTR reporter by ELAVL4. This suggested that either ELAVL4 controls MYCN expression by impairing other miRNAs than miR-17 or secondary, largely miRNA-independent regulation by ELAVL4. In conclusion, these findings suggest IGF2BP1 as a potent, RNA-binding and miRNA-dependent regulator of MYCN expression. Thus, IGF2BP1 likely contributes to the uncoupling of elevated miR-17-92 and MYCN expression.

### The miR-17 Seed Family Is the Main Antagonist of MYCN Expression

**Figure 5:**
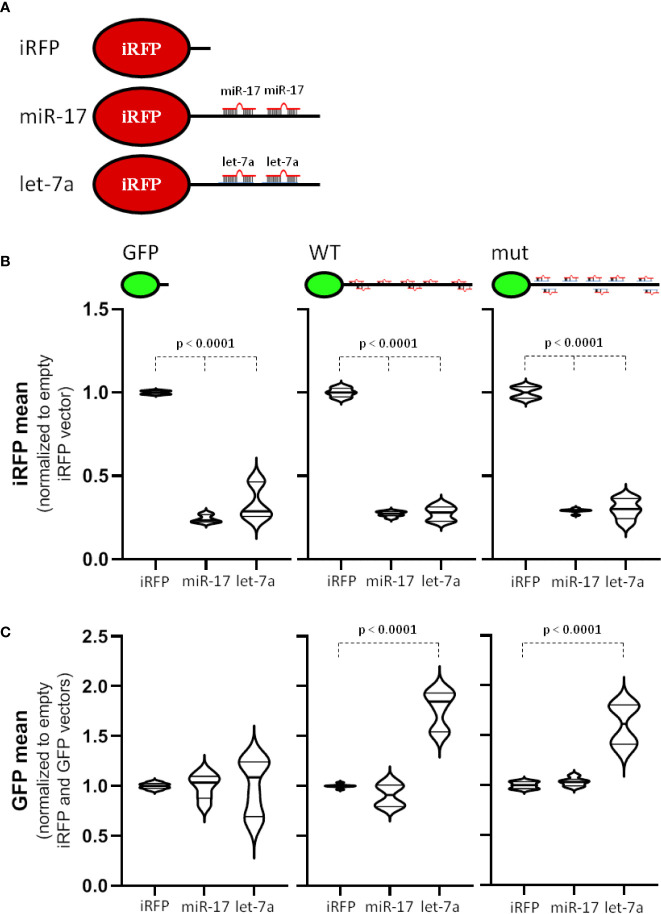
miR-17 is a strong antagonizing miRNA for MYCN 3´UTR. **(A)** Scheme of used iRFP vectors. Control vector (iRFP) contains only a minimal 3´UTR. For miRNA antisense vectors, two perfectly complementary miRNA-targeting sites (miR-17 or let-7a) were cloned behind the iRFP open reading frame. **(B, C)** Transfected BE(2)-C cell were analyzed by flow cytometry. GFP and iRFP double positive cells were analyzed to determine the influence of the MYCN 3´UTR to the antisense reporter **(B)** or the influence of the miRNA antisense reporter to the MYCN 3´UTR **(C)** upon normalization (n = 3). Statistical significance was determined by Student’s t-test (p-values indicated).

Our analyses revealed a strong effect of miRNAs of the miR-17 seed family and potential involvement of let-7 family members in controlling MYCN expression in neuroblastoma. Notably, it was proposed that the MYCN-3’UTR serves as a let-7a sponge (11). Aiming to evaluate the postulated potency of the MYCN-3’UTR in sponging main regulatory miRNAs, we explored the activity of iRFP-fused miR-17 and let-7a antisense reporters (**Figure 5A**) when expressing the aforementioned GFP-reporters (**Figure 3A**). The expression of iRFP from both miRNA antisense reporters was markedly reduced in BE(2)-C cells when co-expressed with the GFP control reporter (**Figure 5B**, left panel), indicating substantial activity of miR-17 and let-7a in BE(2)-C cells. Most notably, however, the activity of both antisense reporters remained largely unaffected when expressing either the wild type or the miRNA mutant MYCN-3’UTR fused to GFP (**Figure 5B**, middle and right panel). This strongly argues against a miRNA sponge effect of the MYCN-3’UTR.

Our miTRAP studies revealed a strong enrichment of miR-17-92 cluster miRNAs with the MYCN-3’UTR, whereas let-7 miRNAs, including let-7a, were only modestly enriched (see **Figure 2** and **Supplementary Table 4**). Thus, to evaluate if regulation of the MYCN-3’UTR by the respective miRNAs is distinct, we evaluated the expression of GFP reporters when expressing the respective iRFP miRNA antisense reporters (**Figure 5C**). Whereas expression of the control GFP reporter remained largely unaffected by the miRNA antisense reporters, the wild type, but also the miRNA mutated (including inactivation of all reported let-7 targeting sites) MYCN-3’UTR reporter showed markedly elevated expression by co-expressing the let-7a antisense reporter. Although requiring further in depth investigation, these findings largely suggest that the let-7-dependent regulation of MYCN expression is largely secondary, but provides no conclusive evidence for a sponging role of the MYCN-3’UTR.

## Discussion

We provide a comprehensive analysis of miRNA expression in primary neuroblastoma. These studies reveal that *MYCN*-amplified (MNA) neuroblastoma is sharply distinguished by the upregulated expression of miRNAs associated with adverse diseases outcome and the downregulation of miRNAs associated with more favorable prognosis. Among sharply upregulated miRNAs are members of the miR-17-92 cluster, which are increased by MYCN at the transcriptional level and impair MYCN expression by feed-back regulation (12, 26, 27). This strongly argues, that elevated MYCN expression in MNA neuroblastoma is not substantially supported by the downregulation of major MYCN-regulatory miRNAs, as previously proposed (10).

In MNA neuroblastoma BE(2)-C cells, expressing both, MYCN protein as well as miR-17-92 at substantial levels, members of the miR-17-92 cluster are the most enriched miRNAs observed in MYCN-3’UTR miTRAP studies. Likewise, we demonstrate striking enrichment of miR-17-92 members with the MYCN-3’UTR also in *MYCN*-driven glioblastoma KNS42 cells. Together with ample evidence for a pivotal role of the miR-17-92 cluster miRNAs in controlling MYCN expression, this indicates that this miRNA family is an essential regulator of MYCN mRNA abundance. However, the co-upregulation of both, MYCN protein and the miR-17-92 cluster miRNAs, in MNA neuroblastoma suggests that miRNA-dependent regulation of MYCN expression is substantially modulated by trans-acting factors controlling MYCN expression *via* the 3’UTR. To this end, we have investigated two RNA-binding proteins (RBPs), previously reported to control MYCN expression in neuroblastoma, ELAVL4 (HuD) and IGF2BP1. Whereas ELAVL4 expression is decreased in MNA neuroblastoma, IGF2BP1 expression is elevated. MNA neuroblastoma is considered an aggressive and de-differentiated disease. In this respect, the downregulation of ELAVL4 is consistent with its proposed role in promoting neural differentiation, as reviewed in (45). On the contrary, IGF2BP1 upregulation in MNA neuroblastoma is supported by previous studies in primary tumors, *MYC/MYCN*-driven transcriptional regulation and conserved oncofetal expression of the protein (13, 14, 46, 47). Notably in this respect, the conserved upregulation or *de novo* synthesis of IGF2BP1 in cancer is primarily observed in progressed, de-differentiated malignancies, as demonstrated for instance in anaplastic thyroid carcinoma (48). MYCN-3’UTR reporter studies provide strong evidence that both proteins modulate MYCN expression in a 3’UTR-dependent manner. This is consistent with the reported role of ELAVL4 in antagonizing downregulation of MYCN expression by miR-17 and recently reported roles in the 3’UTR-dependent enhancement of mRNA translation in neural and neuroblastoma-derived cells (12, 49). However, stimulation of MYCN-3’UTR reporter expression by ELAVL4 remained essentially unaffected by inactivating miR-17 targeting sites. In contrast, the RNA-dependent regulation of MYCN-3’UTR reporters by IGF2BP1 was essentially lost upon inactivation of miR-17 and other miRNA-targeting sites. Together, this provides strong evidence that IGF2BP1 is a potent miRNA antagonist upregulating MYCN mRNA and protein in neuroblastoma. This likely is of pivotal importance in MNA neuroblastoma, where IGF2BP1 probably serves as a *MYCN*-driven antagonist of MYCN-inhibitory miRNAs upregulated in MNA neuroblastoma.

In addition to investigating trans-acting factor promoting MYCN expression in MNA neuroblastoma in a 3’UTR- and miRNA-dependent manner, we also evaluated if the MYCN-3’UTR may serve as a miRNA decoy for MYCN-targeting miRNAs, as previously proposed (11). This was investigated for MYCN-targeting miRNAs upregulated in MNA neuroblastoma, like miR-17-92 cluster miRNAs, or substantially expressed like the let-7 miRNA family. MiTRAP studies indicate that both miRNA families are enriched with the MYCN-3’UTR, although enrichment was substantially pronounced for miR-17-92 cluster miRNAs. MiRNA antisense reporter analyses, however, clearly demonstrate that the MYCN-3’UTR lacks decoy activity, since expression of either reporter remained essentially unchanged when overexpressing the MYCN-3’UTR fused to GFP. Moreover, expression of the miR-17 antisense reporter remained ineffective in changing GFP-MYCN-3’UTR expression providing supportive evidence that neither the antisense nor the native MYCN-3’UTR have miRNA decoy activity. Why the let-7a antisense reporter changed expression of GFP reporters irrespective of let-7 targeting site inactivation remains unclear at present. However, in view of perturbed expression of both reporters, it appears likely that altered GFP reporter expression results from secondary regulation.

In conclusion our studies provide a comprehensive view on the expression of miRNAs in neuroblastoma and provide further insights into the pro-oncogenic role of the RNA-binding protein IGF2BP1 involving positive feed-back regulation with MYCN in neuroblastoma, in particular *MYCN*-amplified (MNA) diseases. These findings reveal new avenues for the treatment of MNA neuroblastoma. In recent studies, we demonstrated that inhibition of IGF2BP1-RNA association by the small molecule BTYNB impairs tumor cell growth *in vitro* and in *xenograft* mouse models (43, 50). Moreover, we recently showed for the first time that circular RNA miRNA decoys directed against the potent oncomiR 21-5p impair tumor cell vitality *in vitro* and tumor growth in *xenograft* models when delivered *via* nanoparticles (51). This highlights new avenues to pursue the targeting of post-transcriptional regulation in cancer, in particular the strongly 3’UTR-dependent regulation of MYCN mRNA in MNA neuroblastoma.

## Data Availability Statement

Presented data have been deposited in NCBI’s Gene Expression Omnibus and are accessible through GEO Series accession number GSE155945 (https://www.ncbi.nlm.nih.gov/geo/query/acc.cgi?acc=GSE155945). Small RNA-seq normalized count data are also available via the R2: Genomics Analysis and Visualization Platform (http://r2.amc.nl; datasets: “Tumor Neuroblastoma - Bell - 97 - tmm - mirbase22”) for interactive use and visualization.

## Ethics Statement

Ethical review and approval was not required for the study on human participants in accordance with the local legislation and institutional requirements. Written informed consent from the participants’ legal guardian/next of kin was not required to participate in this study in accordance with the national legislation and the institutional requirements.

## Author Contributions

The study was conceptualized by SHü, SHa, JS, and DM. Data analyses were performed by DM and SHa. Experiments were performed by SHa, ML, JB, NB, and BB. The manuscript was written by DM, SHa, JS, and SHü. Figures were prepared by DM and SHa. All authors have read and agreed to the published version of the manuscript. All authors contributed to the article and approved the submitted version.

## Funding

The work was partially supported by DFG-funding (RTG1591) to SH and intramural Roux-funding and Monika Kutzner Stiftung to JB.

## Conflict of Interest

The authors declare that the research was conducted in the absence of any commercial or financial relationships that could be construed as a potential conflict of interest.

## Supplementary Material

Supplementary Figure 1 MiRNA enrichment in miTRAP studies is independent of miRNA abundance. The enrichment of miRNAs with the MYCN-3’UTR (compared to MS2 controls) was plotted over the abundance of respective miRNAs in input samples, as determined by miRNAseq. No correlation of miRNA abundance and enrichment was observed by Pearson correlation in indicated cell lines. BE(2)-C: R_P_ = 0.001072; p = 0.9688 and KNS42: R_P_ = 0.002382; p = 0.9306.

Supplementary Figure 2 Expression of IGF2BP1 and ELAVL4 in neuroblastoma. The expression of IGF2BP1 (left panel) and ELAVL4 (right panel) in MNA (92 samples) and nMNA (401 samples) neuroblastoma tumors were analyzed in a public available mRNA-seq data set on R2 database (https://r2.amc.nl; SEQC dataset, GEO ID: GSE49710).
