# Supplementary figures and images for: The MicroRNA Landscape of MYCN-Amplified Neuroblastoma
Source: Front Oncol. 2021 May 7;11:647737. doi: 10.3389/fonc.2021.647737 (PMC8138323; doi:10.3389/fonc.2021.647737)

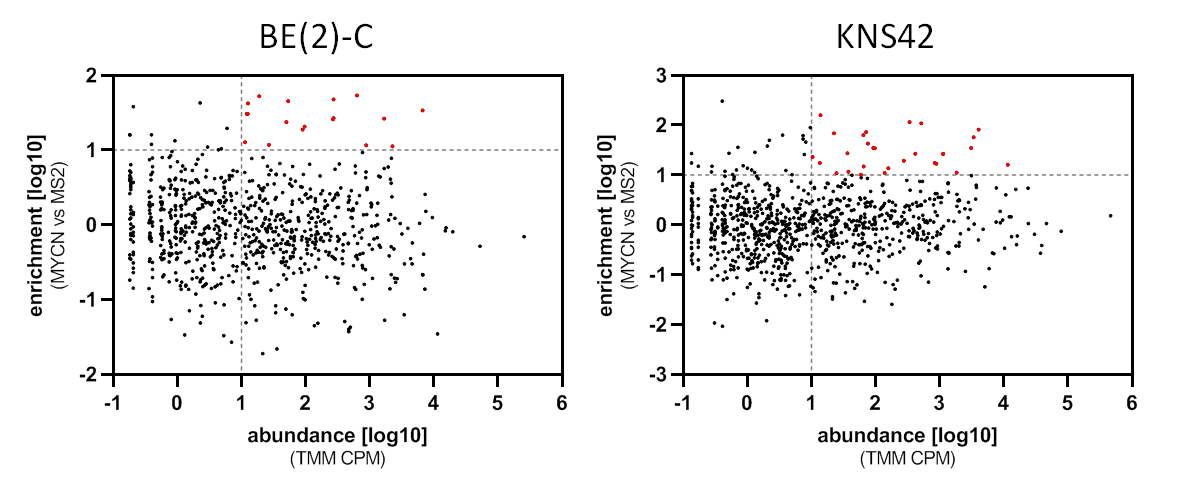

Supplement: Supplementary Figure 1 — MiRNA enrichment in miTRAP studies is independent of miRNA abundance. The enrichment of miRNAs with the MYCN-3’UTR (compared to MS2 controls) was plotted over the abundance of respective miRNAs in input samples, as determined by miRNAseq. No correlation of miRNA abundance and enrichment was observed by Pearson correlation in indicated cell lines. BE(2)-C: RP = 0.001072; p = 0.9688 and KNS42: RP = 0.002382; p = 0.9306. [file Image_1.tif]

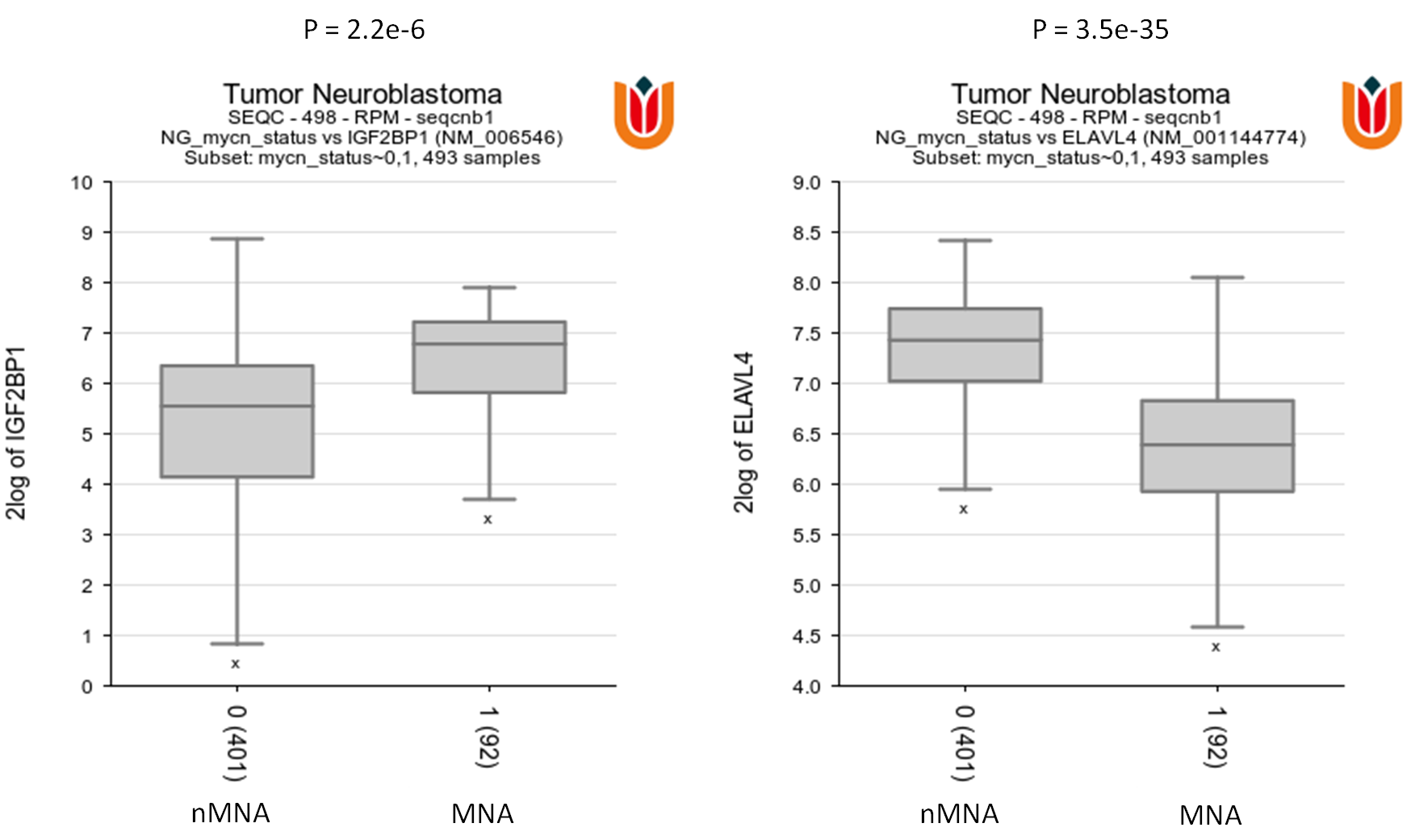

Supplement: Supplementary Figure 2 — Expression of IGF2BP1 and ELAVL4 in neuroblastoma. The expression of IGF2BP1 (left panel) and ELAVL4 (right panel) in MNA (92 samples) and nMNA (401 samples) neuroblastoma tumors were analyzed in a public available mRNA-seq data set on R2 database (https://r2.amc.nl; SEQC dataset, GEO ID: GSE49710). [file Image_2.tif]
